# 

**Published:** 1801-02

**Authors:** 


### TO CORRESPONDENTS.

*The Editors are again obliged to apologize to their Correspondents for the omission of several valuable Communications, and particularly for delaying an account of many Medical Publications, both domeftic and foreign.*

